# CFEM proteins: unique to fungi with essential roles in plant host interactions

**DOI:** 10.3389/ffunb.2026.1866984

**Published:** 2026-09-04

**Authors:** Edgar David Carrillo-Hernández, Nohemi Carreras-Villaseñor, Javier Plasencia, Eric Edmundo Hernández-Dominguez, Diana Sánchez-Rangel

**Affiliations:** 1 Laboratorios de Fitopatología y Biología Molecular, Instituto de Ecología A.C. (INECOL), Red de Estudios Moleculares Avanzados (REMAv), Xalapa, Mexico; 2 Laboratorio de Proteo-metabolómica y Bioquímica de Proteínas, Instituto de Ecología A.C. (INECOL), Red de Estudios Moleculares Avanzados (REMAv), Xalapa, Mexico; 3 Departamento de Microbiología. Centro de Investigación Científica y Educación Superior de Ensenada (CICESE), Ensenada, Mexico; 4 Departamento de Bioquímica, Facultad de Química, Universidad Nacional Autónoma de México, Mexico City, Mexico; 5 Investigador por México-SECIHTI, Xalapa, Mexico

**Keywords:** CFEM domain, effectors, host-pathogen interactions, phytopathogens, immunity suppression

## Abstract

Fungal diseases pose a serious threat to agriculture, forestry, and human health sectors and are major contributors to global economic losses. Plant-pathogenic fungi rely on a wide array of molecules that contribute to pathogenicity and virulence; thus, it is critical to understand their roles in plant-fungal interactions to propose control strategies. Among these, CFEM domain-containing proteins, which are unique to fungi primarily within the phyla Ascomycota and Basidiomycota, have diverse functions in the interaction with the host plant. The CFEM domain is characterized by the presence of eight highly conserved cysteine residues, although the proteins show remarkable structural diversity, leading to three functional groups: I) Secretion proteins, II) GPI-anchored proteins, and III) Pth11-like membrane proteins. Current studies have focused on phytopathogenic species, where these proteins not only act as effectors that disrupt the plant immune response but also play roles in maintaining cell wall integrity, heme acquisition, and infection structure formation. However, the diversity among these proteins, as well as their subcellular localization, suggest novel mechanisms remain to be discovered. Herein, we review the phylogenetic distribution of CFEM domain-containing proteins in various species, the relevance of the cysteine residues, and their classification and functional diversity, aiming to provide an integrated approach for a better understanding of their role in fungal cell physiology as well as in the interaction with the host.

## 1 Introduction

Fungi constitute a remarkably abundant and diverse kingdom, with an estimated 2.2 to 5 million species (Hawksworth and Lücking, 2017; Li et al., 2021). Phytopathogenic fungi are particularly noteworthy due to their ability to infect different tissues —leaves, stems, roots, and fruits— in a wide range of plants (Coque et al., 2020), that results in significant adverse effects on the growth and yield of agriculturally and forestry important species (Davydenko et al., 2018; Dobbs et al., 2021; Y. Peng et al., 2021; Vázquez-Avendaño et al., 2021). Plant-pathogenic fungi exhibit diverse lifestyles that determine their interactions with plants, which are classified into: biotrophs, hemibiotrophs, and necrotrophs (Lo Presti et al., 2015; Pawlowski and Hartman, 2016). Biotrophs require living cells for nutrient uptake and suppress plant immunity through the secretion of multiple effector molecules that contribute to manipulating the host immune response, while necrotrophs mainly produce toxins and hydrolytic enzymes, causing host cell death to obtain nutrients necessary for their development and reproduction (Fei and Liu, 2023; Liao et al., 2022). In contrast, hemibiotrophic fungi are considered particularly versatile due to their ability to establish a biotrophic interaction during the early stages of colonization, and then switch to a necrotrophic lifestyle (Fei and Liu, 2023; Liao et al., 2022; Lo Presti et al., 2015).

Regardless of their lifestyle, plant pathogenic fungi produce effector molecules that disrupt plant host defenses through multiple mechanisms. Effectors are defined as small molecules, RNAs, or proteins produced and secreted by a pathogen that suppress or neutralize host defense responses and metabolic functions, thereby facilitating infection and colonization (Kainat et al., 2025; Luti et al., 2020). To better recognize their role in virulence and to design strategies to prevent and tackle fungal infections, it is crucial to identify relevant effector molecules in fungal pathogens and understand their mode of action. Studies on the secretome of filamentous fungi report that it constitutes approximately 4% to 15% of the total number of fungal coding genes (Feldman et al., 2020). Most of these proteins are enzymes, such as proteases, lipases, cellulases, pectinases, and cutinases, but also proteins lacking catalytic activity. Among the latter are Cysteine-Rich Proteins (CRP), which constitute a significant component of the fungal secretome and have been implicated in various fungal–plant pathosystems (Pellegrin et al., 2015; Stergiopoulos and De Wit, 2009). Cysteine (Cys) is one of the least frequent amino acids (aa), with an average content of 1.5% aa in proteins. In contrast, CRPs are characterized by a higher Cys content, ranging from 3% to 5% of the total aa composition (Shcherbakova et al., 2016; Zhao et al., 2021). Cys is highly sensitive to the local cellular pH and redox microenvironment which facilitates sulfur oxidation states from −1 to +4. Its nucleophilic thiol side chain (-SH) mediates transition metal coordination and redox signaling cascades (Percio et al., 2024).

When two thiols groups from adjacent Cys residues are in proximity and properly oriented, they undergo oxidation to form covalent intra- and intermolecular disulfide bonds, contributing significantly to protein folding. The disulfide bonds formation stabilize proteins, allowing them to resist, for example, degradation by plant-produced proteases as a defense mechanism (Bulaj, 2005; Dombkowski et al., 2014). Moreover, in antioxidant enzymes such as glutathione peroxidase and thioredoxin, whose activity depends on Cys, the thiol group acts as a reducing agent, neutralizing reactive oxygen species (ROS) (Netto et al., 2007; Castro et al., 2021). Because Cys residues are highly susceptible to oxidation by ROS, they are present in proteins that sense redox changes in the cellular environment and regulate the oxidative stress response (Duan et al., 2017; Yan, 2014). In fungi, CRPs have diversified to perform distinct functions: 1) cerato-platanin proteins are effectors in pathogenic species (Gaderer et al., 2014; Luti et al., 2020; Zhao et al., 2021), 2) hydrophobins act as water-repellents on the surface of filamentous fungi (Sunde et al., 2008; Wösten and Scholtmeijer, 2015), and 3) CFEM (Common in the Extracellular Fungal Membrane) domain-containing proteins (henceforth referred to as CFEM proteins), have multiple functions (Kulkarni et al., 2003). The CFEM domain is characterized by the presence of eight Cys and constitutes the main focus of this review, which has recently garnered significant interest in understanding their function and relevance in various fungal species (Hong et al., 2024; Li et al., 2025; H. Liu et al., 2024; S. Liu et al., 2024). Herein, we discuss their role as key players in plant pathogenesis and highlight the significant knowledge gaps remaining.

## 2 CFEM domain-containing proteins or CFEM proteins

**Figure 1:**
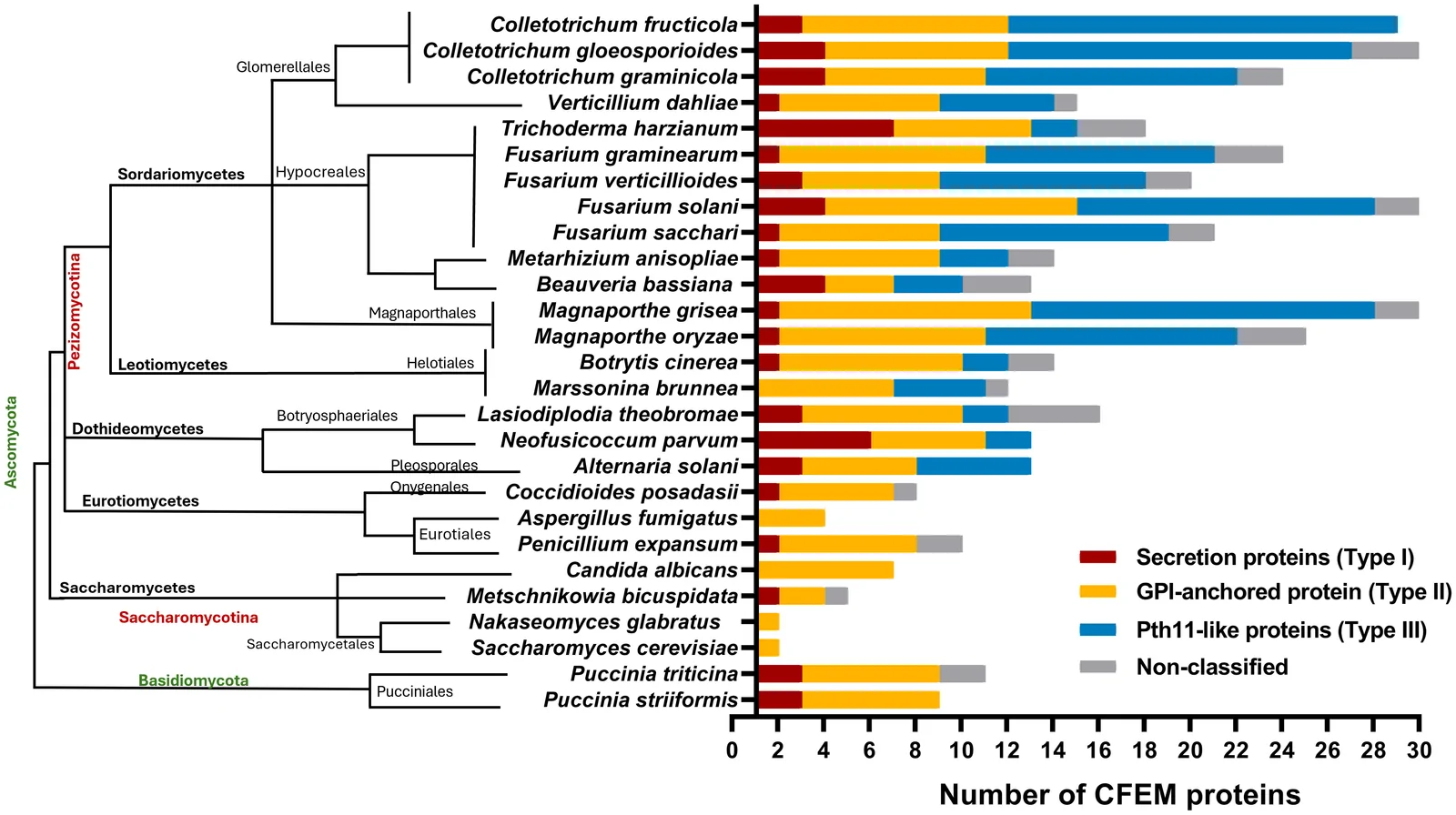
Phylogenetic analysis and distribution of CFEM proteins across species. The cladogram illustrates the phylogenetic relationships of 27 species based on data obtained from the MycoCosm. A panel on the right lists the species containing CFEM proteins together with the total number identified in each genome. The graph highlights the variability in the number of CFEM proteins among species, emphasizing phylogenetic differences and suggesting potential functional disparities in their distribution. The distribution of CFEM proteins is further categorized into three classes (Types I–III) according to the proposed classification, and their abundance in each species is shown. The phylogenetic tree was obtained directly from the MycoCosm database, which provides a reference phylogeny for fungal species. CFEM proteins were classified based on structural features: the presence of GPI anchoring using the Big-PI Fungal Predictor, transmembrane helices using TMHMM, and predicted subcellular localization using DeepLoc-2.0.

According to Pfam, the CFEM domain (PF05730) is a fungal-specific Cys-rich motif that plays an important role in fungal pathogenesis. The CFEM domain was first identified by Kulkarni and co-workers in 2003, in the extracellular protein ACI1, produced by the rice blast fungus *Magnaporthe grisea* during appressorium development. Through a Yeast Two-Hybrid (Y2H) assay, they identified the interaction between the adenylate cyclase MAC1 and ACI1, which is characterized by an N-terminal CFEM domain (Kulkarni et al., 2003; Kulkarni and Dean, 2004; Deng and Dean, 2008). The CFEM domain (SMART: SM00747; InterPro: IPR008427) consists of approximately 60 aa residues with a specific consensus sequence defined by: PxC[A/G]x2Cx8–12Cx1–3[x/T]Dx2–5CxCx9–14Cx3–4Cx15–16C (Kulkarni et al., 2003). Structurally, the CFEM domain exhibits a unique and highly conserved helical-basket fold composed of six α-helices stabilized by four disulfide bonds formed by eight conserved Cys residues (Nasser et al., 2016). CFEM domain proteins constitute a highly conserved family, with most members possessing a signal peptide ranging from 16 to 24 aa and mature proteins ranging from 80 to 600 aa (Peng et al., 2022; Qiu et al., 2024; Shang et al., 2024). Interestingly, genomic analyses of diverse organisms, including animals, plants, and fungi, have determined that the CFEM domain is unique to fungi and phylogenetic evidence reveals that this domain evolved from a common ancestor, indicating that proteins harboring this domain belong to a single large family. Furthermore, these findings indicated that is restricted to the phyla Ascomycota and Basidiomycota, thus suggesting its origin in the most recent common ancestor of these phyla (Kulkarni et al., 2003; Zhang et al., 2015) (**Figure 1**).

## 3 Classification of CFEM domain-containing proteins

Since the identification of the CFEM domain, numerous studies have characterized these proteins across various species using phylogenetic analyses and bioinformatics tools. These methods rely on the prediction of structural features, such as signal peptides, GPI-anchor sites, and transmembrane domains, to classify CFEM proteins as previously reported in *Colletotrichum graminicola* (Gong et al., 2020), *Fusarium graminearum* (Zuo et al., 2022), and *Fusarium verticillioides* (Li et al., 2025), among others. However, whether distinct clusters exist within this unique CFEM protein family remains an open question. To address this, we performed CLuster ANalysis of Sequences (CLANS; Frickey and Andrei, 2004) on 10,000 CFEM domain-containing sequences retrieved from the InterPro database. Analyses across multiple *p* value thresholds (1e -10, -20 or -200) revealed a global network connecting all sequences (**Supplementary Figure 1**). This connectivity reflects the presence of the conserved CFEM motif across all analyzed proteins, supporting their classification onto a single family based on sequence similarity. Nonetheless, several minor subgroups comprised low percentages of total sequences, whereas the most cohesive groups were enriched for sequences predicted to contain transmembrane domains (**~**2,200 sequences). Furthermore, CLANS analysis showed approximately 1,400 sequences remained unclustered, indicating that sequence-based subdivision requires further refinement. This limitation reinforces the rationale for a functional classification. Therefore, this review focuses on a three-group functional classification supported by experimental evidence.

In this regard, Feng et al. (2024) proposed a phylogenetic classification that categorized CFEM proteins into three main groups. Type I CFEM proteins have a secretory signal peptide lack transmembrane site and they play an important role in pathogenesis because they act as effectors modulating plant immunity (Feng et al., 2024; Shang et al., 2024). Type II CFEM proteins have a signal peptide and are associated with GPI anchoring (Feng et al., 2024; Ikezawa, 2002). This group of proteins can localize in both the cell wall and the plasma membrane, where they integrate a heme acquisition and transport process, mainly characterized in *Candida albicans* (Nasser et al., 2016; Roy and Kornitzer, 2019). Similarly, the entomopathogenic filamentous fungus *Beauveria bassiana* colonizes its host from the cuticle to the hemolymph, where GPI-anchored proteins are involved in heme acquisition (Peng et al., 2022; Zhang et al., 2025). Furthermore, this group plays crucial roles in pathogenicity, cell wall stability, conidial production, and heme group acquisition (Chen et al., 2021; Nasser et al., 2016; Vaknin et al., 2014; Zhu et al., 2017).

**Figure 2:**
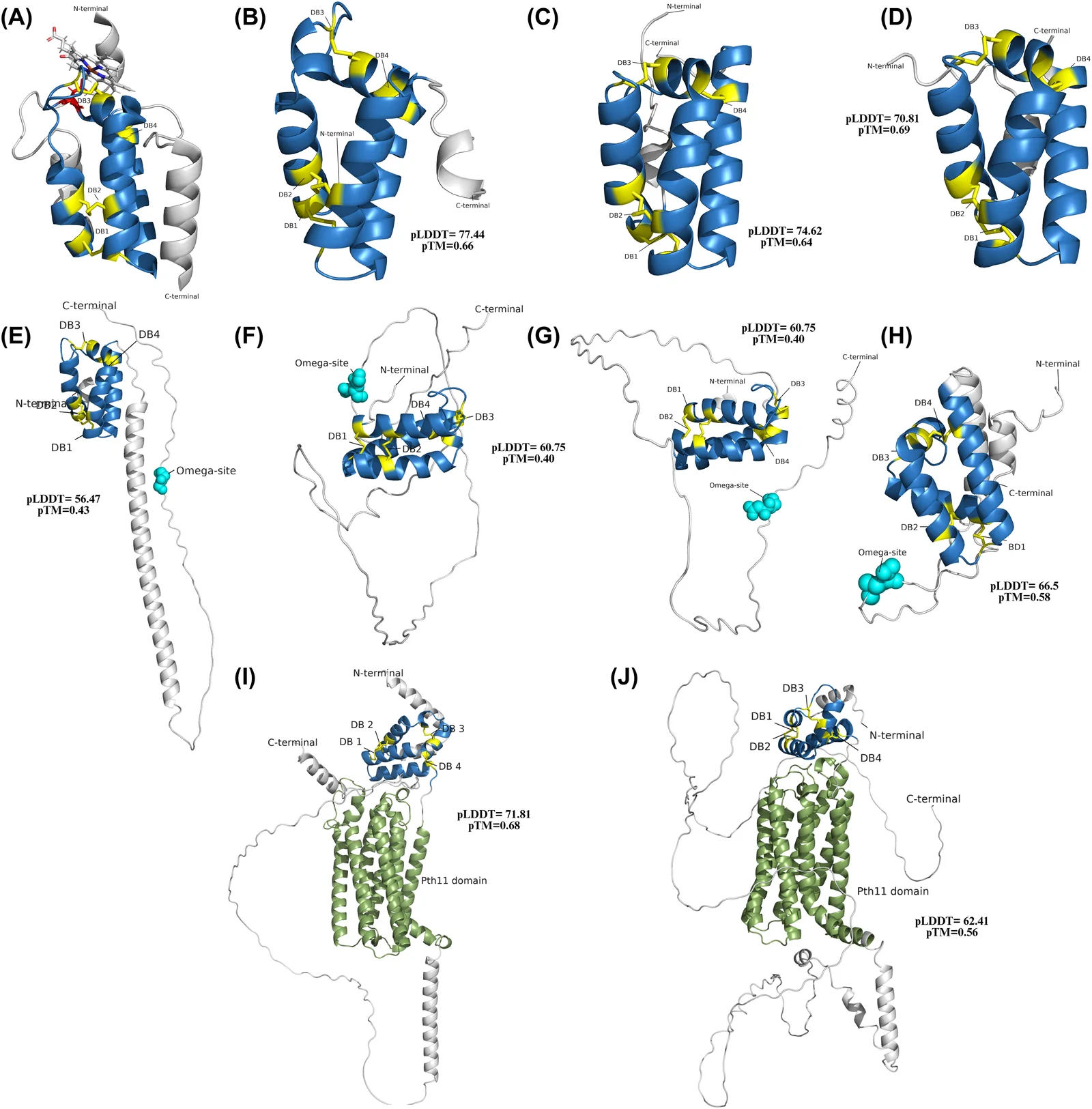
Predicted three-dimensional structures of representative CFEM proteins. Panels **(A–J)** show individual three-dimensional models were obtained from AlphaFold DB, selected based on high mean predicted Local Distance Difference Test (pLDDT) and predicted TM-score (pTM) values. The proteins are organized according to the three CFEM types. **(A–D)** Type I Secretion proteins (ID Uniprot): Csa2 (PDB: 4Y7S; https://www.rcsb.org/structure/4Y7S), VdSCP77 (G2X658), MaCFEM88 (A0A0D9P8F2) and FgCFEM11 (I1RIG7). **(E–H)** Type II GPI-anchored proteins: AsCFEM6 (A0AA52HNH8), CfmB (Q4WMA6), FgCFEM1 (I1REI8) and FgCFEM5 (I1RET0). **(I–J)** Type III Pth11-like proteins: WISH (G5EI24) andPth11 (Q9Y784). In all models, the CFEM domain adopts the characteristic “helical-basket” fold and is shown in cartoon representation (sky blue), with the four predicted disulfide bonds (DB1–DB4) displayed as yellow sticks. In Type II proteins, residues corresponding to predicted GPI-anchor attachment sites are indicated as cyan spheres. Type III proteins contain transmembrane regions separating the extracellular CFEM-containing domain from intracellular regions and C-terminal cytoplasmic tails. Predicted signal peptides were omitted from Type I and Type II proteins for visualization purposes. Structural models were retrieved from the AlphaFold DB and not generated *de novo*. Protein selection was based on pLDDT and pTM confidence scores. Structural visualization and figure editing were performed using PyMOL and BioRender.

Type III proteins are Pth11-like proteins with seven transmembrane domains that act as membrane receptors involved in host signal perception (Feng et al., 2024; Kou et al., 2017; Kulkarni et al., 2005). Finally, the proteins in the “non-classified” group do not exhibit the putative characteristics that define the proposed categories and are therefore included in this group. However, future functional, structural, and phylogenetic studies could provide additional information that would allow for a more precise classification of these proteins. **Figure 2** illustrates the three-dimensional models of the three types of CFEM proteins obtained from AlphaFold DataBase (Bertoni et al., 2025).

**Figure 3:**
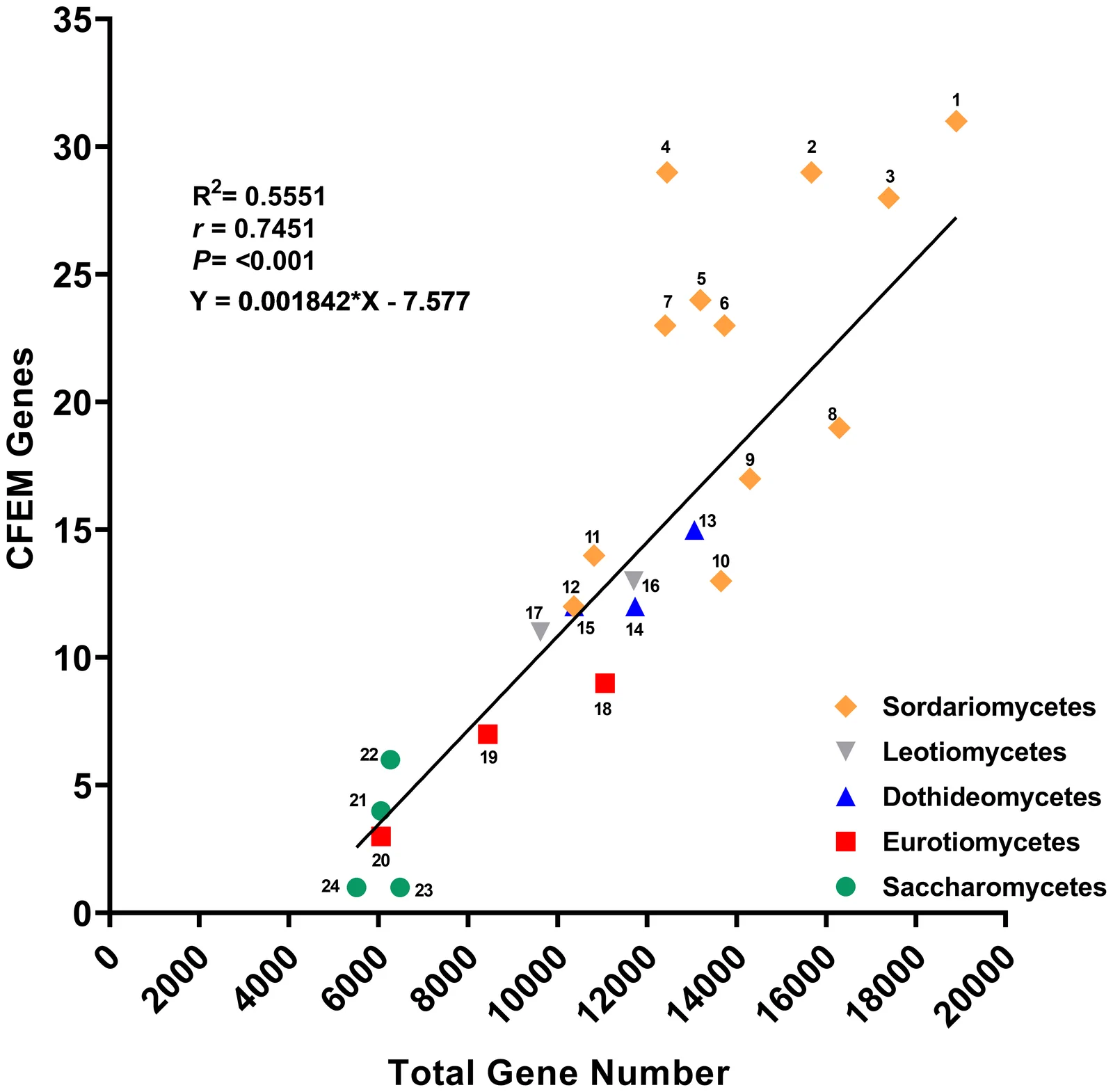
Relationship between total gene number and CFEM gene number. Graphic showing the relationship between total gene number and CFEM gene number across 24 fungal species. Each numbered point represents one species: 1, *F. solani*; 2, *C. gloeosporioides*; 3, *C.fructicola*; 4, *M. grisea*; 5, *M. oryzae*; 6, *F. graminearum*; 7, *C. graminicola*; 8, *F. verticillioides*; 9, *T. harzianum*; 10, *M. anisopliae*; 11, *V. dahliae*; 12, *B. bassiana*; 13, *L. theobromae*; 14, *A. solani*; 15, *N. parvum*; 16, *B. cinerea*; 17, *M. brunnea*; 18, *P. expansum*; 19, *C. posadasii*; 20, *A. fumigatus*; 21, *M. bicuspidata*; 22, *C. albicans*; 23, *S. cerevisiae*; and 24, *N. glabratus*. Pearson’s correlation analysis revealed a significant positive association between CFEM gene number and total gene number (r = 0.7451, P < 0.001). The Pearson correlation coefficient, P-value, regression equation, and coefficient of determination (R²) are indicated in the plot. Total gene numbers were obtained from reference genome annotations available at the National Center for Biotechnology Information (NCBI). Correlation analysis and simple linear regression were performed using GraphPad Prism version 8.

So far, studies on CFEM proteins have been conducted in 27 species across various fungal groups, including entomopathogens, phytopathogens, and human pathogens (**Figure 1**). Our bioinformatic analysis identified over 350 genes encoding CFEM proteins in these organisms, with approximately 60 proteins functionally characterized via transient expression assays, mutant generation, biochemical analysis, and protein–protein interaction assays, among other methods (**Table 1**). For this review, we used this dataset to expand the comparative analysis previously published by Zhang et al. (2015). We investigated a potential correlation between the copy number of CFEM genes and the total number of genes in the genomes of across 25 Ascomycota species. These genomes ranged from 5,508 (*Nakaseomyces glabratus*; Saccharomycetes) to 18,900 (*Fusarium solani*; Sordariomycetes) with an average of 11,723 genes. Most species contain between 10,000 and 14,000 annotated genes. A robust positive correlation was found between the number of CFEM genes and the total count (Pearson’s correlation: *r* = 0.7451, p < 0.001), suggesting that species with greater overall genetic content tend to have a higher number of CFEM genes (**Figure 3**).

**Table 1:** Comprehensive overview of CFEM-containing proteins in phytopathogenic fungi.

ID uniprot	CFEM protein	Type	Species	Evidence of role in fungi-host interaction	Reference
E3Q595	CgCFEM6	Type I	*Colletotrichum graminicola*	Suppression of induced cell death identified by transient coexpression with BAX in *N. benthamiana* leaves.	(Gong et al., 2020)
E3Q704	CgCFEM7				
E3Q7L5	CgCFEM8				
E3QD10	CgCFEM9				
E3QTK8	CgCFEM15				
UPI0006352007	ThCFEM1		Trichoderma harzianum*	Suppression of induced cell death identified by transient coexpression with XEG1 in *N. benthamiana* leaves.	(Todd et al., 2025)
A0A180G6V3	PTTG_08198		*Puccinia triticina*	Promotes cell death and triggers ROS accumulation from transient expression in *N. benthamiana* through *Agrobacterium* infiltration.	(Zhao et al., 2020)
A0A5N5DH84	LtCFEM1		*Lasiodiplodia theobromae*	Induced a local yellowish phenotype and cell death from transient expression in *N. benthamiana* through *Agrobacterium* infiltration, respectively	(Peng et al., 2021)
A0A5N5CVT0	LtCFEM4				
G2X659	VdSCP76		*Verticillium dahliae*	Suppress the host immune response, characterized by callose deposition, ROS accumulation, and induction of cell death.	(Wang et al., 2022)
G2X658	VdSCP77				
K1XVG1	MbCFEM1		*Marssonina brunnea*	Suppresses the host immune response and enhances *Fusarium proliferatum* infection in *N. benthamiana* leaves	(Qian et al., 2022)
K1WG73	MbCFEM6				
K1XW16	MbCFEM8				
K1XT82	MbCFEM9				
A0A0D9P8F2	MaCFEM88		*Metarhizium anisopliae*	Transient expression elicits a hypersensitive response in *N. benthamiana* leaves	(Cai et al., 2022)
A0ACC0ECY0	PstCFEM1		*Puccinia striiformis f.* sp. *tritici*	Suppress the host immune response, characterized by callose deposition, ROS accumulation, and induction of cell death.	(Bai et al., 2022)
–	PstCFEM2			PstCFEM2 blocks TaHA2 phosphorylation mediated by the TaCIPK9 kinase. This increases apoplastic acidification and promotes stomatal opening, facilitating fungal colonization by the fungus.	(Y. Zhang et al., 2026)
–	Fs06761		*Fusarium sacchari*	Contributes to virulence during interaction with the sugarcane cultivar ‘Zhongzhe 1’ and suppresses host immune responses	(Huang et al., 2023)
–	Fs08184				
–	Fs13617				
L2FGQ8	CfEC12		*Colletotrichum fructicola*	Suppression of cell death through interaction with MdNIMIN2, a regulator of NPR1 that plays a positive role in SA–dependent plant immunity in *Malus domestica.*	(Shang et al., 2024)
–	Fs08184		*Fusarium sacchari*	Virulence is closely associated with two specific residues in CFEM domain: aspartic acid (Asp, D) and phenylalanine (Phe, F).	(Hong et al., 2024)
–	Fs10706				
–	Fs13617				
G2X283	VdTRP		*Verticillium dahliae*	A non-canonical CFEM protein involved in host cell death induction through phosphatidylserine mediated interaction.	(Liu et al., 2024)
L2FGQ8	CgCFEM1		*Colletotrichum gloeosporioides*	Gene deletion caused reduced pathogenicity, which was associated with alterations in spore morphology, conidiation, and appressorium development.	(Feng et al., 2024)
W7MII6	FvCFEM12		*Fusarium verticillioides*	Suppresses BAX-induced cell death in *N. benthamiana*. Interaction with ZmWAK17ET.	(Li et al., 2025)
I1RIG7	CFEM11 (Synonym FgCFEM11)		*Fusarium graminearum*	Immune responses mediated by ZmWAK17 in *Zea mays* are suppressed through interactions with ZmLRR5 and ZmWAK17ET. Increased mRNA levels during infection in wheat coleoptiles	(Zuo et al., 2022; Chen et al., 2021)
I1RGH5	FgCFEM23			Increased mRNA levels during infection in wheat coleoptiles	(Chen et al., 2021)
R1GA23	NpCysRP5	Type II GPI-anchored	*Neofusicoccum parvum*	Exhibiting a significant increase of *NpCysRP5* expression during interaction with *Liquidambar styraciflua.*	(Vázquez-Avendaño et al., 2021)
J4KM55	BbCFEM7		Beauveria bassiana*	Involved in virulence during interaction with *Galleria mellonella* larvae. Additionally, genes of the BbCFEM family exhibit a functional compensation mechanism.	(Peng et al., 2022, 2025; Zhang et al., 2025)
J4UHQ3	BbCFEM8				
E9EVT4	MaCFEM85		Metarhizium anisopliae*	Transient expression causes a hypersensitive response in *N. benthamiana* leaves and interacts with MsWAK16 proteins, leading to the induction of plant resistance.	(Cai et al., 2022, 2023)
I1REI8	CFEM1		*Fusarium graminearum*	Immune responses mediated by ZmWAK17 in *Zea mays* are suppressed through interactions with ZmLRR5 and ZmWAK17ET.	(Zuo et al., 2022)
I1RET0	CFEM5				
A0AA52HNH8	AsCFEM6		*Alternaria solani*	Elicits a hypersensitive response in multiple hosts, and gene deletion results in reduced virulence.	(Qiu et al., 2024)
A0AA49QAE1	AsCFEM12				
–	CgCsa		*Colletotrichum gloeosporioides*	Gene deletion resulted in reduced colony diameter, conidia production, and germination. In addition, pathogenicity assays revealed decreased virulence, which was associated with defects in infection-related structures (appressorium).	(Liu et al., 2024)
Q8J0Q3	ACI1		*Magnaporthe grisea*	The first protein with a CFEM domain identified in *M. grisea*, can interact with the adenylate cyclase MAC1, which is involved in cAMP production, a key regulator of appressorium development.	(Kulkarni et al., 2003; Kulkarni and Dean, 2004)
Q4WLB9	CfmA		Aspergillus fumigatus*	The individual and combined deletion of the three CFEM proteins showed alterations in cell wall stability and structure, without causing defects in growth or germination. Additionally, these deletions did not reduce the virulence of *Aspergillus fumigatus*.	(Vaknin et al., 2014)
Q4WMA6	CfmB				
Q4WNE1	CfmC				
A0A384JUL4	BcCFEM1		*Botrytis cinerea*	Deletion of BcCFEM1 results in reduced virulence. Moreover, its transient expression induces chlorosis, suggesting a potential role as an elicitor in plant pathogen interaction.	(Zhu et al., 2017)
—	FsCFEM1		*Fusarium solani*	The alkaloid cepharanthine (CEP) can bind to FsCFEM1 through the binding sites Tyr724, Thr748, and Gly861. This interaction affects signal transduction and membrane stability.	(Yuqing Wang et al., 2025b)
A0A0A2KHR5	PeCFEM5		*Penicillium expansum*	Both genes are induced during the early hours of infection in apples. Individual deletion affected spore development, decreased tolerance to cell wall stress agents, and reduced the virulence in apples and pears. In addition, deletion of both genes suppressed the accumulation of patulin (PAT), a mycotoxin harmful to human health.	(Yanling Wang et al., 2025)
A0A0A2JWQ9	PeCFEM8				
G5EI24	WISH	Type III Pth11-like	*Magnaporthe oryzae*	Associated with host surface recognition and appressorium morphogenesis, as well as vegetative hyphal autolysis, regulation of surface hydrophobicity, and maintenance of cell wall integrity.	(Sabnam and Roy Barman, 2017)
G4N244	Pth11		*Magnaporthe grisea*	Transducer of signals present on the host surface, functioning as an upstream regulator of the cAMP-dependent signaling pathway. This pathway is essential for the differentiation and development of the appressorium.	(Kulkarni et al., 2005; Kou et al., 2017; Xu et al., 2017)

*Trichoderma harzianum: beneficial fungus*, Beauveria bassiana* and *Metarhizium anisopliae:* entomopathogenic fungi and *Aspergillus fumigatus* is primarily recognized as a saprotrophic fungus.

The accession numbers for the proteins with a ‘–’ were not found in the UniProt databases.

**Figure 4:**
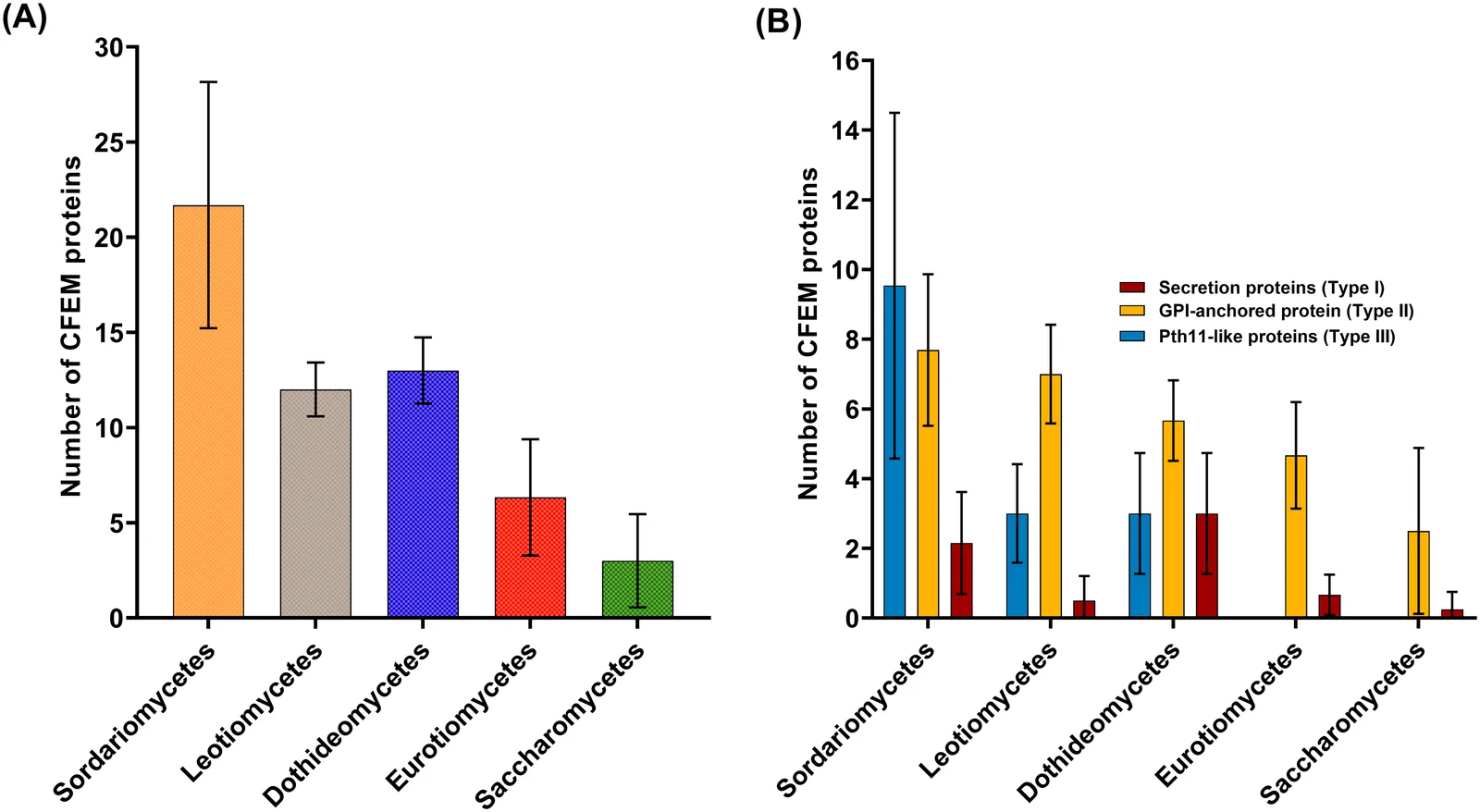
Comparison and classification of CFEM protein types in Ascomycota classes. Bar plots showing the distribution of CFEM proteins grouped by fungal taxonomic class. **(A)** Average number of CFEM proteins per species within each class. **(B)** For each CFEM type, the number of proteins identified in each species was averaged separately within its corresponding taxonomic class. Bar plots and standard deviations were generated using GraphPad Prism version 8.

Despite the inherent bias of focusing solely on species with functionally characterized CFEM proteins, we found that the species analyzed belonging to the phylum Ascomycota fall into five main classes: Sordariomycetes, Leotiomycetes, Dothideomycetes, Eurotiomycetes, and Saccharomycetes (**Figure 4**). Among these, Sordariomycetes exhibited the highest average number of CFEM proteins (21.69 ± 6.47), whereas Eurotiomycetes and Saccharomycetes showed lower abundances, with averages of 6.3 ± 3.05 and 3 ± 2.4 CFEM proteins, respectively (**Figure 4A**). Although species within the latter classes possess key enzymes involved in the degradation of plant components such as cellulose and pectin, these enzymes are primarily associated with saprophytic and decomposition processes (McLaughlin and Spatafora, 2015; Ownley and Trigiano, 2016). In contrast, Sordariomycetes, Leotiomycetes, and Dothideomycetes include numerous highly aggressive phytopathogens, which may account for the higher number of CFEM proteins observed in these groups (**Figure 4A**) (Goodwin, 2013; Zhang et al., 2015; Maharachchikumbura et al., 2016).

Within plant pathogenic fungi, in Sordariomycetes class, such as *C. gloeosporioides*, the causal agent of anthracnose disease in various crops, *Colletotrichum graminicola*, the causal agent of anthracnose disease of maize, *F*. *graminearum*, the organism responsible Fusarium Head Blight (FHB) in wheat, barley and other grains, and *F. sacchari*, that provoke sugarcane wilt, multiple CFEM proteins have been reported with an average of approximately 25 proteins per species (Feng et al., 2024; Gong et al., 2020; Zuo et al., 2022; Huang et al., 2023; Chen et al., 2021). In these species, the average number of effector proteins (CFEM Type I) is 2.15 ± 1.43, while GPI-anchored proteins (CFEM Type II) average 7.69 ± 2.17, and Pth11-like proteins (CFEM Type III) average 9.53 ± 4.96 (**Figure 4B**). In contrast, within Dothideomycetes species, such as *Lasiodiplodia theobromae* and *Neofusicoccum parvum*, both pathogens belong to the Botryosphaeriaceae family, causing canker, dieback, and fruit rot diseases in a wide range of woody plants, and *Alternaria solani*, the causal agent of early blight in solanaceous crops, an average of 13 CFEM proteins have been identified (Peng et al., 2021; Qiu et al., 2024). In this class, the number of effector proteins is 3 ± 1.73, GPI-anchored proteins is 5.6 ± 1.15, and Pth11-like proteins is 3 ± 1.41 to (**Figure 4B**). Finally, it should be noted that the species analyzed for this review belonging to the Eurotiomycetes and Saccharomycetes classes lacked CFEM Type III proteins, which are particularly relevant due to their role as membrane receptors involved in host signal perception and the activation of cAMP (Cyclic Adenosine Monophosphate)-dependent signaling pathways (Kou et al., 2017; Kulkarni et al., 2005; Xu et al., 2017). This remarkable difference in the abundance and distribution of CFEM proteins among fungal classes suggests significant diversity, indicating the possible existence of specific adaptive strategies in phytopathogens to interact more efficiently with their hosts, thus favoring virulence and suppression of immune responses.

## 4 Type I secretion proteins

This group includes CFEM proteins with a signal peptide (SP) and lacking transmembrane domains, which are categorized as secretion proteins. CFEM proteins in this group have been studied across various pathosystems and localized to the plant apoplast, cytosol, plasma membrane, and nucleus, depending on their specific function, and highlighting their role as effectors (Cai et al., 2022; Gong et al., 2020; Qian et al., 2022). Proteins within this group are small, typically comprising less than 200 amino acids, and exhibit high diversity, with low sequence identity both within the CFEM domain and across the full-length sequence. In *F. graminearum*, 23 CFEM proteins have been identified, 11 of which are predicted to be secreted. Within this subset, CFEM domain sequence identity ranges from 13% to 34%, whereas full-length protein sequence identity ranges from 12% to 32% (Zuo et al., 2022). In terms of structure, the available information is limited; most models have been generated using homology-based methods with tools such as PHYRE2 and SWISS-MODEL. These models are primarily based on the unique crystallized structure of the CFEM protein Csa2 from *C. albicans* (Cai et al., 2022; Gong et al., 2020; Qian et al., 2022). In this structure, the CFEM domain adopts a distinctive “helical basket” configuration with six alpha helices stabilized by disulfide bonds formed between the eight Cys residues (Nasser et al., 2016; **Figure 2A**). Structurally, Csa2 harbors an extended N-terminal loop to display a heme molecule over a flat hydrophobic platform on its CFEM domain. The complex is stabilized by two key residues: an axial iron ligand, Asp80, and a novel stacking interaction with Tyr36. As the first experimentally resolved CFEM structure, Csa2 provides structural blueprints vital for understanding type I CFEM proteins and their conserved functional features. Finally, as the authors indicate, Csa2 is involved in iron acquisition, functioning as part of a strategic mechanism that pathogenic fungi use to overcome the host nutritional immunity (Nasser et al., 2016). The effector function and their roles in virulence have been documented by biochemical and genetic evidence. Transient expression assays in *N. benthamiana* have shown that CFEM proteins can induce a hypersensitive response (HR), a mechanism involving programmed cell death associated with plant immunity. These CFEM proteins include MaCFEM88 (**Figure 2C**) from *Metarhizium anisopliae*, a soil-borne entomopathogenic fungus (Cai et al., 2022); g11649 from *Falciphora oryzae*, a beneficial root endophytic fungus (Dai et al., 2024); and LtCFEM1 and LtCFEM4 from the broad-host-range phytopathogen *L. theobromae* (Peng et al., 2021).

Moreover, Type I CFEM proteins impact on several elements of the plant host immune system, such as the suppression of programmed cell death induced by BAX (Bcl-2-associated X protein) or INF1 (elicitin infestin 1) genes. This ability has been shown through transient coexpression assays performed in *N. benthamiana* for various CFEM proteins: *F. verticillioides* (FvCFEM12), *C. fructicola* (CfEC12), *C. graminicola* (CgCFEM6, CgCFEM7, CgCFEM8, CgCFEM9, and CgCFEM15), *F. sacchari* (Fs06761, Fs08184, and Fs13617), *V. dahliae* (VdSCP76 and VdSCP77; **Figure 2B**) and *Puccinia striiformis* (PstCFEM1) (Li et al., 2025; Shang et al., 2024; Gong et al., 2020; Huang et al., 2023; Wang et al., 2022; Bai et al., 2022). To evaluate the role of disulfide bridge formation in CFEM proteins of *V. dahliae*, targeted mutagenesis was performed, substituting Cys for alanine (C→A) in VdSCP76 and VdSCP77, since alanine (Ala) lacks the thiol group. Such changes result in the loss of their ability to suppress VdEG1-induced cell death, highlighting the potential role of disulfide bridges in the regulation of immunity (Wang et al., 2022; Zuo et al., 2022). Moreover, CfEC12, PstCFEM1, VdSCP77, and VdSCP76 suppress the plant immune response by inhibiting cell wall reinforcement, specifically the deposition of callose, a β-(1,3)-D-glucan polysaccharide that acts as a structural barrier to limit pathogen spread (Shang et al., 2024; Bai et al., 2022; D. Wang et al., 2022; Jones and Dangl, 2006; Qi et al., 2019; Wang et al., 2021).

Although *N. benthamiana* remains widely used, potential baseline defense responses or specific folding requirements for Cys-rich motifs should be considered (Beritza et al., 2024). To address this, complementary approaches such as mutant generation have also been employed to further elucidate the functions of these proteins. For example, in *F. verticillioides*, deletion of *FvCFEM12* affects hyphal morphology and reduces cell wall stress tolerance (Li et al., 2025). In terms of virulence, the mutant strain produces smaller lesions on susceptible maize stalks. Similarly, complete deletion of *CfEC12*, especially the SP-truncated version, reduced lesion diameters on *Malus domestica* leaves infected with *C. fructicola* (Shang et al., 2024). Together, these findings demonstrate that both complete protein and effector secretion are critical determinants of pathogenicity. Moreover, when cotton seedlings were inoculated with conidia of the Δ*VdSCP76* and Δ*VdSCP77* strains of *V. dahliae*, colonization decreased after 21 dpi compared to the WT strain. This was evidenced mainly by reductions in fungal biomass and discoloration phenotypes in longitudinal sections of the shoots (Wang et al., 2022).

A non-canonical CFEM protein has recently been identified in *V. dahliae*, characterized by the presence of only six Cys residues in the CFEM domain. Despite this difference, structural analyses suggest that the CFEM domain adopts a three α-helix conformation, like those observed in CFEM proteins from species such as *M. grisea, Botrytis cinerea, Fusarium sporotrichioides, C. albicans*, and *Coccidioides immitis* (H. Liu et al., 2024). Additionally, a specific proline-rich motif has been identified within this protein, consisting of the sequence IPGGCNPAHPGPGSCP, which is present in a four-repeat tandem repeat central region. Due to this distinctive feature, the protein has been designated as *V. dahliae* tetrapeptide tandem repeat protein (VdTRP). Functionally, the CFEM domain is crucial for membrane attachment in cotton through its interaction with phosphatidylserine (PS), a lipid essential for regulating cell surface charge and protein localization. Once anchored to the membrane, VdTRP is involved in inducing plant host cell death (H. Liu et al., 2024).

While previous studies hypothesized that some CFEM proteins act as effectors, recent evidence has elucidated their direct interactions with specific plant targets. The stripe rust fungus (*P. striiformis*) secretes the PstCFEM2 effector to promote wheat susceptibility. It achieves this by manipulating TaHA2 (a plasma membrane H^+^-ATPase) and the kinase TaCIPK9, resulting in apoplastic acidification. Under normal conditions, TaCIPK9 phosphorylates TaHA2 at Ser-933, inducing a conformational change that suppresses enzyme activity and maintains apoplastic pH homeostasis. During infection, PstCFEM2 competitively binds to the C-terminal domain of TaHA2, preventing TaCIPK9-mediated phosphorylation. The hyperactive TaHA2 pumps excess protons into the apoplast, lowering extracellular pH. This acidic environment facilitates fungal colonization and triggers increased stomatal opening, allowing the pathogen to thrive and spread (Zhang et al., 2026).

**Figure 5:**
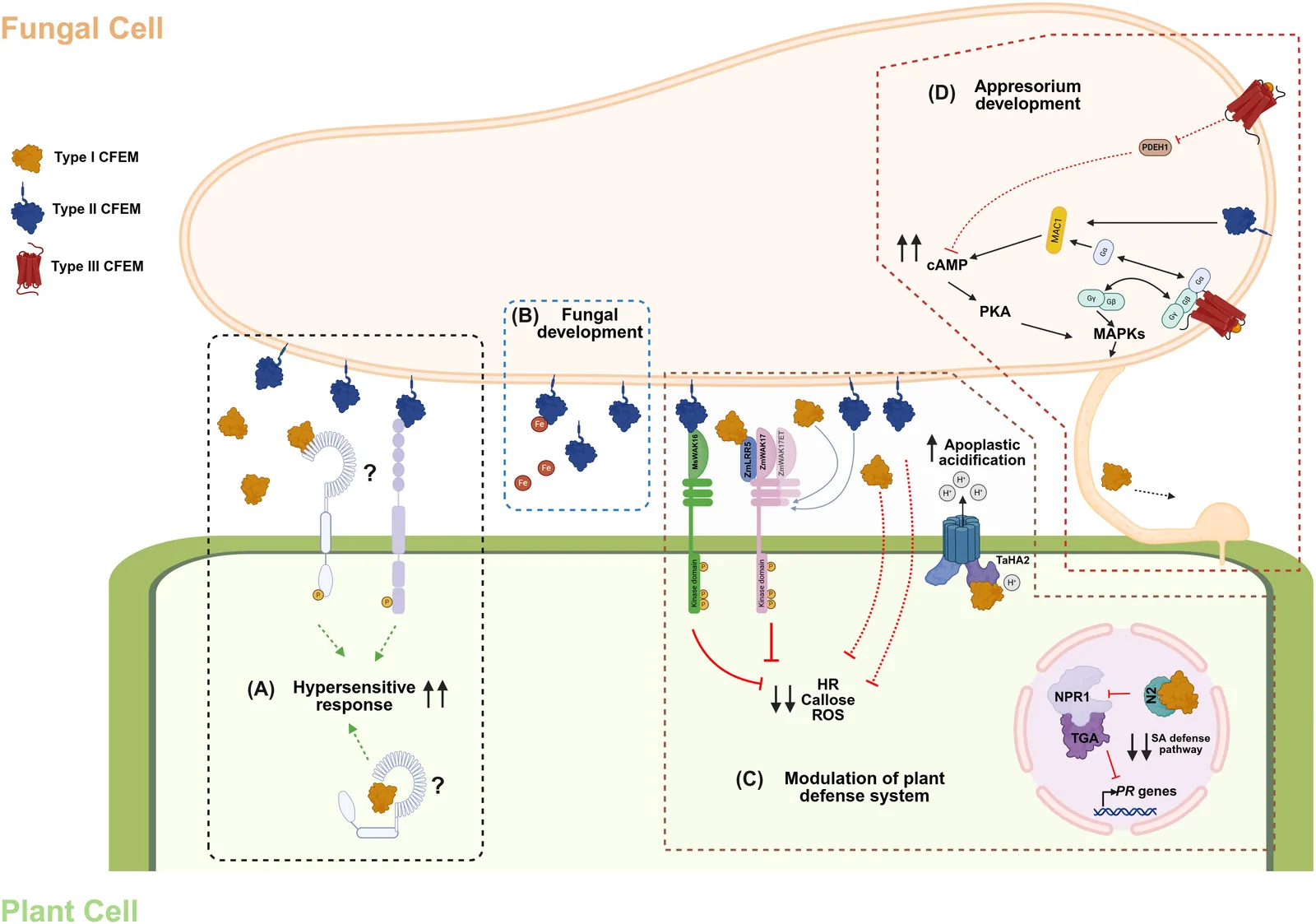
Overview of four distinct roles of CFEM proteins in phytopathogenic fungi. **(A)** Induction of Hypersensitive response: Type I and II CFEM-containing proteins elicit HR, as reported for LtCFEM1 and LtCFEM4 (Type I) from *L. theobromae*, which induce chlorosis and necrosis, respectively. Similarly, AsCFEM6 and AsCFEM12 (Type II) from the necrotrophic species *A. solani* can induce cell death symptoms. It is currently unknown which type of plant receptor (transmembrane or cytoplasmic) interacts with CFEM proteins. **(B)** Fungal Development: This function is primarily mediated by Type II proteins anchored to the plasma membrane and cell wall. Proteins such as CfmA–CfmC in *A. fumigatus* are involved in stabilizing the cell wall against external stressors. Iron acquisition is also critical for fungal development; BbCfem7 in *B. bassiana* (an entomopathogenic fungus) is required for fungal growth and is essential for virulence in *Galleria mellonella* larvae. **(C)** Modulation of plant defense system: CFEM domain proteins modulate plant immunity mainly through direct interaction with host kinases and key regulators. For instance, *Fusarium* spp. Type II proteins CFEM1, CFEM5 and Type I CFEM11 (synonym FgCFEM11) and FvCFEM12 inhibit the signal transduction of the receptor kinase ZmWAK17, by interaction with ZmWAK17ET and ZmLRR5, which is necessary for activating defense-related genes. Similarly, the Type II protein MaCFEM85 of *M. anisopliae* interacts with MsWAK16. Regarding key regulators, *C. fructicola* secretes and delivers CfEC12 (Type I) into the cell nucleus and binds to MdNIMIN2 (N2), a positive regulator of plant immunity, thereby preventing its interaction with MdNPR1, targeting the plant SA defense pathway. A third example involves the Type I PstCFEM2 protein, which inhibits TaCIPK9-mediated phosphorylation of TaHA2. This proton pump drives apoplastic acidification and promotes stomatal opening, thereby facilitating colonization by *P. striiformis*. Finally, various Type I CFEM proteins, such as Fs13617 and Fs08184 from *F. sacchari*, VdSCP76 and VdSCP77 from *V. dahlie*, as well as CgCFEM6–CgCFEM9 from *C. graminicola*, have been implicated in the suppression of the immune response induced by elicitors such as BAX and INF1. **(D)** Appressorium development: This function has been mainly linked to *Magnaporthe* species, in which Type III proteins such as Pth11 and WISH are implicated. Pth11 acts as a signal transducer within a highly conserved cAMP/PKA and MAPK signaling pathways, initiated by the recognition of environmental signals that activate heterotrimeric G protein subunits (Gα, Gβ, and Gγ). WISH (Type III) serves as a negative regulator of PDEH1, resulting in interruption of cAMP-mediated G-protein signaling. In addition, the Type II protein ACI1from *M. grisea* has been shown to interact with the adenylate cyclase MAC1, which is involved in cAMP production. Finally, the Type I protein CgCFEM1 from *C. gloesporoides* has been reported to be important for appressorium development. All these processes involve the coordinated participation of various proteins, transcription factors, and other molecules that contribute to the execution of each function. Green lines indicate activation/induction. Red lines indicate repression. Dash lines indicate that the molecular process is unknown. Gα, Gβ, and Gγ, protein subunits; MAC1, Adenylate cyclase; cAMP, Cyclic Adenosine Monophosphate; PKA, protein kinase A; PDEH1, phosphodiesterase; N2, NIM1-INTERACTING protein; NPR1, NONEXPRESSER OF PR GENES1; TGA, TGA-bZIP transcription factors; ROS, Reactive Oxygen Species; PR genes, Pathogenesis-Related genes. Created with BioRender.

*F. graminearum* and *F. verticillioides*, two relevant maize pathogens, produce CFEM effector proteins to hijack maize Wall-Associated Kinases (WAKs), resulting in the attenuation of the host immune response. In *F. graminearum*, Zuo et al. (2022) used Y2H and bimolecular fluorescence complementation (BiFC) assays to identify and confirm protein interactions. They found that several apoplastic CFEM proteins, including FgCFEM11, interact with key immune-related proteins in *Zea mays*, most notably the ZmWAK17 kinase (**Figures 2D**, **5C**). This pathogenic association is uniquely mediated through two host intermediary proteins: ZmWAK17ET, an alternatively spliced isoform of ZmWAK17, and ZmLRR5 (a leucine-rich repeat protein). By binding to these intermediaries, CFEM effectors successfully suppress ZmWAK17-induced hypersensitive cell death, weakening the plant’s immune response and heavily increasing susceptibility to *F. graminearum* infection (Zuo et al., 2022). As the authors discussed alternative splicing yields ZmWAK17 variants with opposing roles in host-fungal interactions through a fine-tuned regulatory mechanism. Specifically, the full-length ZmWAK17 promotes resistance, whereas the truncated ZmWAK17ET isoform drives CFEM-mediated susceptibility. In *F. verticillioides*, the study identifies specifically FvCFEM12 as a dual-function effector that drives virulence by suppressing plant immunity and maintaining fungal cell wall integrity. By interacting with the maize kinase ZmWAK17, this effector facilitates the transition from endophytism to pathogenicity, causing significant host susceptibility (Li et al., 2025).

In the *C. fructicola-Malus domestica* pathosystem, the CfEC12 protein interacts with MdNIMIN2, a positive regulator of plant immunity, blocking its interaction with MdNPR1. This disruption weakens NPR1-dependent immune responses, which are crucial for the salicylic acid (SA)-mediated regulation of defense genes in apples. The authors concluded that MdNPR1 is essential for resistance to *C. fructicola*, thus facilitating host infection (Shang et al., 2024).

Collectively, these findings (e.g., interaction with WAKs by *F. graminearum*/*verticillioides*, NIMIN2 by *C. fructicola*, and TaHA2 by *P. striiformis*) indicate that CFEM effectors act as adaptive virulence agents. They enable phytopathogenic fungi to infect plant systems at multiple levels by exploiting various defense signaling pathways. While *Fusarium* spp. effectors primarily operate at the cell surface to block receptors; *P. striiformis* CFEM effectors also interfere with initial defense layers, and *Colletotrichum* effectors penetrate the host to directly disrupt internal hormonal signaling hubs. This suggests that CFEM effectors have evolved functional diversity to target different cellular compartments and defense layers, adapting to the specific infection strategies of each fungal pathogen.

## 5 Type II GPI-anchored proteins

CFEM proteins in this group possess a N-terminal signal peptide, which directs them to the endoplasmic reticulum (ER), and also a motif sequence at the C-terminal that is involved in peptide cleavage and anchoring to the plasma membrane via the glycosylphosphatidylinositol (GPI); alternatively, they may localize to the cell wall through linkages with β-glucans (Fujita and Kinoshita, 2012; Kinoshita and Fujita, 2016; Essen et al., 2020; Kinoshita, 2020; Vogt et al., 2020).

Given that GPI-anchored CFEM proteins vary greatly in length, ranging from 150 aa, such as ACI1 in *M. grisea*, to 1018 aa, such as CSA1 in *C. albicans*, they likely adopt multiple structural conformations, which may account for the diverse biological functions they perform in the encoding species (Deng and Dean, 2008; Klis et al., 2009). Therefore, this section on Type II CFEM proteins highlights their roles in maintaining cell wall integrity, facilitating iron acquisition, and eliciting or modulating host immunity.

### 5.1 Cell wall integrity

Functionally, GPI proteins in filamentous fungi have been mainly associated with cell wall remodeling and maintenance (Zhu et al., 2017; Ouyang et al., 2019; Samalova et al., 2020). This function has also been observed in GPI-anchored CFEM proteins of *Aspergillus fumigatus* (Vaknin et al., 2014). Although single (Δ*CfmA*, Δ*CfmB*, Δ*CfmC*), double (Δ*CfmA*/*CfmB*, Δ*CfmA*/*CfmC*, Δ*CfmB*/*CfmC*), and triple (ΔTriple-Cfm) deletion mutants showed no significant differences in radial growth or germination percentage, triple mutants are more sensitive to the cell wall stress agents Congo Red (CR) and Calcofluor White (CFW). These results suggest that CFEM proteins of *A. fumigatus* are involved in the stabilization and maintenance of cell wall integrity (Vaknin et al., 2014). Similarly, deletion of yeast CFEM proteins such as Ccw14 in *S. cerevisiae* (Mrsa et al., 1999) and Ssr1 in *C. albicans* (Plaine et al., 2008) caused hypersensitivity to CFW, indicating defects in cell wall integrity.

### 5.2 Iron acquisition

In addition to their role in cell wall integrity, the CFEM domain GPI proteins in *C. albicans* —Rbt5, Pga7, and Csa2— are also specialized in heme acquisition (Sorgo et al., 2013; Zhang et al., 2015; Nasser et al., 2016; Weissman et al., 2021). To perform this function, a conserved aspartate Asp residue (Asp80 in Csa2; **Figure 2A**, Asp72 in Rbt5, and D63 in Pga7) within the “helical basket” configuration of the CFEM domain acts as an axial ligand for the iron atom of heme (Kuznets et al., 2014; Nasser et al., 2016). Substitution of the conserved Asp residue in Rbt5 and Pga7 by Ala abrogates the binding to the heme group, confirming the key role of this residue in the heme acquisition process (Kuznets et al., 2014). Regarding transport of the heme group, Rbt5, locates in the outer layer of the cell wall, acts as an intermediary in its acquisition through two possible pathways: 1) the secreted Csa2 captures the ferric iron ion (Fe³^+^) released from methemoglobin and then transfers it to Rbt5 (Nasser et al., 2016; Roy et al., 2022); or 2) through direct interaction with methemoglobin (Roy and Kornitzer, 2019). Once acquired, the heme group is then transferred to Pga7, located in the inner cell wall and plasma membrane, where it receives the heme from Rbt5 (Roy and Kornitzer, 2019; Roy et al., 2022). Thus, heme transport toward the plasma membrane proceeds via one of two pathways: Csa2 → Rbt5 → Pga7 or Rbt5 → Pga7 (Nasser et al., 2016; Roy and Kornitzer, 2019; Kornitzer and Roy, 2020). Current evidence indicates that heme acquisition is not exclusive to yeasts (Srivastava et al., 2014; Nasser et al., 2016; Roy and Kornitzer, 2019; S. Liu et al., 2024), as this capacity has also been found in CFEM-GPI proteins from phytopathogenic and entomopathogenic fungi.

*C. gloeosporioides* synthesizes CgCsa, a CFEM protein structurally similar to Csa2 (Liu et al., 2024). Molecular docking studies suggest that CgCsa can bind Fe³^+^ ions through the conserved iron-binding site at the Asp residue. This function was further supported experimentally by analysis of intracellular iron content, which showed that the ΔCgCsa strain had lower iron levels than to the WT strain. Consequently, ΔCgCsa showed down-regulation of genes related to iron transport, including the multicopper oxidase FET3 and the MFS (Major Facilitator Superfamily) siderophore iron transporter (CgIron2 and CgIron3). Such alterations in iron acquisition reduced both mycelial growth and conidial production. However, such phenotypes were restored to levels comparable to the WT strain by supplementing the medium with sufficient exogenous Fe³^+^.

Recently, progress has been made in elucidating the mechanisms of iron acquisition in *B. bassiana* and its potential competition with *Enterococcus mundtii* for this resource, which is part of the microbiome of the host, *Spodoptera litura*, a nocturnal moth. BbCFEM7 stands out as a key factor in this competition, as its deletion significantly increases the abundance of *E. mundtii*, which has a highly efficient iron uptake system. This interaction was evidenced by quantifying Fe³^+^ levels in the hemolymph of larvae infected with the WT, Δ*BbCFEM7*, and Δ*BbCFEM7*::*BbCFEM7* strains, both in the presence and absence of *E. mundtii*. Co-inoculation led to a marked decrease in Fe³^+^ levels, confirming the high capacity for iron absorption. Consequently, the increase of *E. mundtii* during interaction with Δ*BbCFEM7* was associated with a pronounced reduction in fungal virulence. Moreover, its excessive proliferation induced metabolic dysregulation in the insect gut and hemolymph, altering metabolite levels of fatty acids, organic acids, and indoles, which are considered key indicators of the host’s intestinal health (Peng et al., 2025; X. Zhang et al., 2026).

Furthermore, during infection, *B. bassiana* conidia adhere to the insect cuticle and degrade it to penetrate and proliferate in the host. In this process, the fungal plasma membrane may undergo structural changes, such as alterations in membrane curvature. BbCFEM7 has been identified as positively contributing to this process. This was corroborated by Δ*BbCFEM7* mutant strains, which exhibited defects in germ tube curvature and reduced virulence in insects. Moreover, the complementation strain restores curvature, penetration, and germination of conidia (Peng et al., 2026).

### 5.3 Elicitation and modulation of host immunity

While GPI-anchored CFEM proteins show broad functional divergence and have fundamental roles in maintaining cell wall structure and stability, studies have also focused on a distinct subset of these proteins that exhibit effector-like functions. Due to their presence on the fungal cell surface, these proteins can potentially interact with plant host proteins, thereby modulating immune responses either through suppression or activation.

In the necrotroph *B. cinerea*, the GPI-anchored protein BcCFEM1 gene is upregulated during infection of bean leaves, and when it is transiently expressed in *N. benthamiana*, it induces chlorosis symptoms. Deletion mutant strains (Δ*BcCFEM1*) display reduced conidial production and decreased their virulence in bean leaves (Zhu et al., 2017). Also, the mutant strain shows susceptibility to osmotic and cell wall stress assays, suggesting that BcCFEM1 contributes to tolerance against harsh environmental conditions (Zhu et al., 2017). Similarly, AsCFEM6 (**Figure 2E**) and AsCFEM12 from *A. solani* triggered HR not only in *N. benthamiana* but also in other species, including cabbage, eggplant, tomato, and sweet viburnum (Qiu et al., 2024). Deletion of these genes results in reduced lesion size and altered conidiation, leading to the overproduction of smaller conidia. However, mutant strains showed increased resistance to mancozeb, chlorothalonil, and propineb, all three fungicides used to control early blight in Solanaceae species. Finally, AsCFEM6 and AsCFEM12 may be involved in the biosynthesis of 1,8-dihydroxynaphthalene (DHN) melanin, a polymer of importance in stringent growing conditions, which is deposited in the cell wall and provides protection against osmotic and cell wall stresses (Qiu et al., 2024).

*Marssonina brunnea* is a hemibiotrophic forest pathogen that causes leaf spot in *Populus* spp., and the genes *MbCFEM1*, *MbCFEM6*, *MbCFEM8*, and *MbCFEM9* are highly expressed during host infection. Transient expression of these proteins in *N. benthamiana* leaves suppresses BAX/INF1-mediated cell death and increases its susceptibility to *F. proliferatum* infection (Qian et al., 2022). Similarly, in the secretome of the *Trichoderma harzianum*–*Pseudocercospora fijiensis* interaction, a Type II CFEM protein (ThCFEM1) was identified, and upon its transient expression in *N. benthamiana* leaves, it suppresses cell death induced by the *Phytophthora sojae XEG1* gene (glycosyl hydrolase family 12) (Todd et al., 2025). In *Penicillium expansum*, a postharvest pathogen of apple, nine CFEM proteins were identified. Among them, PeCFEM5 and PeCFEM8 were associated with fungal development, sporulation, suppression of host cell death induced by BAX and pathogenicity (Wang et al., 2025a). Deletion of these genes results in reduced lesion area and decay rate in apple fruits. A decrease in virulence correlated with 56% and 86% reductions in the mycotoxin patulin in infected tissues with Δ*PeCFEM5* and Δ*PeCFEM8*, respectively. These effects were further supported by downregulation of genes involved in patulin toxin biosynthesis, including *PatC*, *PatH*, *PatL*, and *PatN* in the mutant strains (Wang et al., 2025a).

As mentioned above, during *F. graminearum* and *Zea mays* interaction, the pathogen uses Type I and Type II proteins to counteract the programmed cell death (HR) response induced by the host’s cell wall-associated kinases (WAKs). *F. graminearum* GPI-anchored proteins, CFEM1 (**Figure 2G**) and CFEM5 (**Figure 2H**), specifically interact with the maize proteins ZmLRR5 and ZmWAK17ET, thereby promoting an indirect interaction with ZmWAK17 (Zuo et al., 2022; **Figure 5C**). These protein-protein interactions suppress the hypersensitive ZmWAK17-mediated cell death response. Furthermore, it was determined that targeted mutagenesis, by replacing Cys with Ala in CFEM1, disrupted its binding with ZmWAK17ET, indicating that the CFEM domain is crucial in mediating such interaction (Zuo et al., 2022). Another identified interaction occurs between the MaCFEM85 protein of the entomopathogenic fungus *Metarhizium anisopliae* and the MsWAK16 kinase of *Medicago sativa*. This interaction triggers an early defense response involving the generation of ROS, which activate several signaling pathways, including those involved in plant hormone synthesis, leading to increased jasmonic acid (JA) and decreased SA levels (Cai et al., 2023). Similarly, in *P. expansum*, transient coexpression of PeCFEM5 and PeCFEM8 with BAX for 12 hours reduced SA levels and increased JA levels, suggesting that these CFEM proteins modulate the plant immune response by promoting JA-mediated signaling while suppressing SA-dependent defenses (Wang et al., 2025a).

Based on the evidence, GPI-anchored CFEM proteins have been characterized in line with their essential role in cell wall organization, maintenance, and integrity, supporting the hypothesis that this role is their most likely evolutionary function. But they have also been found in recent years to be functionally diverse, involved in processes like heme acquisition, modulation or elicitation of host immune responses. Regarding the composition of the different CFEM protein groups, the repertoire of CFEM Type I and CFEM Type III varies considerably among the different classes of Ascomycota analyzed. In contrast, CFEM Type II proteins maintain a relatively constant proportion of the total number of CFEM proteins (**Figure 4B**). Altogether, these observations highlight CFEM Type II as one of the most functionally conserved groups within the CFEM family.

## 6 Type III PTH11-like proteins

Type III CFEM proteins comprise a group of G protein-coupled receptors (GPCRs) characterized by an extracellular N-terminal domain, which contains the CFEM domain, seven transmembrane domains, and a cytoplasmic C-terminal domain, which likely interacts with the G-protein complex (Jiang et al., 2024; Kou et al., 2017; Xu et al., 2017; Kulkarni et al., 2005, **Figure 2J**). This group, originally defined by the Pth11 protein from *M. grisea*, is classified as a novel subclass of non-canonical GPCRs specific to ascomycetes (DeZwaan et al., 1999; Kou et al., 2017; Kulkarni et al., 2005). This set of proteins has been identified exclusively in species belonging to Dothideomycetes, Lecanoromycetes, Xylonomycetes, Orbiliales, and Pezizales, all within the subphylum Pezizomycotina (Liu et al., 2025). Notably, no orthologs have been reported in other fungal lineages or in other eukaryotic organisms, indicating restriction to the ascomycete lineage (Kou et al., 2017; Kulkarni et al., 2005). Accordingly, this class of proteins has undergone significant evolutionary expansion, particularly in phytopathogenic species (Jiang et al., 2024).

Experimental evidence from model species of the genus *Magnaporthe* indicates that Pth11 or Pth11-like proteins participate in the recognition of extracellular signals, which trigger conformational changes and activate intracellular signaling cascades, including the cAMP/PKA pathway and the mitogen-activated protein kinase (MAPK) cascade, thereby promoting the formation of infection structures such as the appressorium (Jiang et al., 2024; Kou et al., 2017; Sabnam and Roy Barman, 2017). Although specific studies on Pth11-like proteins and their function remain limited, they can be inferred from their structural and topological similarity to classical GPCRs, as this characteristic architecture is recognized as key for signal recognition and transduction processes (Kou et al., 2017; Kulkarni et al., 2005; Sabnam and Roy Barman, 2017).

Similar to GPCRs, Pth11-related proteins function as receptors, that sense extracellular signals such as hydrophobicity, surface rigidity, and the presence of cutin, but their physiological ligands remain largely unknown (Kou et al., 2017; Sabnam and Roy Barman, 2017). Pth11 acts as a cell surface receptor that recognizes external signals and initiates intracellular signaling cascades, particularly the cyclic AMP (cAMP)-dependent pathway. Upon activation, the intracellular domain of Pth11 interacts with a heterotrimeric G protein. It acts as a guanylate exchange factor (GEF), promoting the exchange of GDP for GTP in the Gα subunit. This process promotes the dissociation of the complex, generating a Gα-GTP subunit and a Gβγ dimer. The Gα-GTP subunit, in its active state, activates the cAMP-PKA signaling pathway, while the Gβγ dimer participates in activating the mitogen-activated protein kinase (MAPK) pathway (**Figure 5D**). Collectively, the perception of host-derived signals and their subsequent transduction are critical for appressorium differentiation and development (Kou et al., 2017; Kulkarni et al., 2005; Xu et al., 2017; Zhang et al., 2024).

Among this new subclass of non-canonical GPCRs retaining the CFEM domain, the WISH (Water Wettability, Infection, Surface Sensing, and Hyper-conidiation) protein of *M. oryzae* stands out due to its multiple functions and pleiotropic effects (Sabnam and Roy Barman, 2017; **Figure 2I**). It participates in mycelial growth, cell autolysis, maintenance of cell wall integrity, conidiogenesis, and appressorium development, the latter being essential for fungal pathogenicity, like Pth11. Infection assays on rice leaves revealed that the Δ*WISH* mutant was unable to produce typical rice blast lesions. This loss of virulence is attributed to impaired detection of hydrophobic surfaces, which directly affects appressorium differentiation; however, no phenotypic changes were observed following exposure to cutin monomers or cAMP. Such alterations in the recognition of external signals or potential ligands highlight the importance of WISH in diverse processes of signal perception and transduction (**Figure 5D**). Collectively, these findings suggest that WISH acts as a key regulator of infection-related morphogenesis by integrating multiple signals necessary for appressorium development (Sabnam and Roy Barman, 2017).

Although research on this group of transmembrane proteins, including Pth11, WISH, and other Pth11-like proteins, remains limited, portions of the signaling pathway associated with these receptors have been described. Their importance in the perception of environmental cues, appressorium formation, and pathogenicity has been demonstrated; however, functional characterization studies have focused primarily on species within the genus *Magnaporthe*. Recently, the identification of Pth11-like proteins in *Colletotrichum* (Gong et al., 2020; Shang et al., 2024) and *Fusarium* (Chen et al., 2021; Li et al., 2025) has been reported. Nevertheless, their biological function and potential involvement in the formation of infection structures, such as the appressorium, remain to be fully elucidated. Consequently, it remains uncertain whether the signal perception and transduction mechanisms characterized in *Magnaporthe* functionally conserved in other phytopathogenic species.

## 7 Conclusions and future directions

More than two decades have passed since the first identification of CFEM as an exclusive and highly conserved domain of fungi and increasing evidence has revealed its relevance to multiple aspects of fungal biology. Previous studies have focused on the evolutionary history of the CFEM domain uncovering potential links between the number of CFEM protein abundance and virulence (Zhang et al., 2015). Still, the driver of its gene duplication or expansion remains unclear. Our sequence clustering analysis confirms that the CFEM family is globally connected and unified by a conserved motif, yet it reveals substantial heterogeneity and a high proportion of unclustered sequences. These findings highlight the limitations of sequence-based classification and suggest that future studies should incorporate more comprehensive analyses to uncover additional conserved motifs and structural features within CFEM-containing proteins.

Additionally, analysis of the phylum Ascomycota reveals a positive correlation between CFEM gene content and total genomic size/gene number (**Figure 3**). Species within Sordariomycetes have the highest number of genes and the most extensive repertoire of CFEM proteins. The differences in CFEM repertoires among Ascomycota classes may reflect divergence associated with ecological niches, host ranges, and lifestyles. This pattern is evident when comparing entomopathogenic and phytopathogenic fungi, which face distinct biological challenges during infection and colonization of their hosts. In insects, fungi must overcome physical barriers, such as the cuticle, as well as immune responses mediated by the Toll pathway and the activity of hemocytes (Qu and Wang, 2018; W. Zhang et al., 2024). Plants, on the other hand, possess highly specialized defense mechanisms (PTI: Pattern-Triggered Immunity and ETI: Effector-Triggered Immunity) capable of recognizing and responding to fungal infection (Stergiopoulos and De Wit, 2009; Lo Presti et al., 2015). This complexity in host-pathogen interactions may be associated with the expansion and functional diversification of CFEM proteins.

Currently, CFEM domain is defined by a conserved consensus sequence, including eight Cys residues and key residues (proline and aspartic acid), which together support a characteristic helical basket conformation (**Figure 2**). In concordance with previous classification (Feng et al., 2024), this review highlights how differences in protein such as GPI anchors modifications or transmembrane domains and consequently cellular localization contribute to functional specialization. Type I secretion proteins function as effectors capable of eliciting or suppressing host immune responses. In contrast, Type II GPI-anchored proteins exhibit greater functional diversity, as they may be essential for cell wall integrity, participate in heme acquisition and transport, or even modulate the host’s immune response. Type III PTH11-like proteins are primarily involved in signal transduction and the regulation of appressorium development. Understanding how other domains or motifs and post translational modifications drive functional diversification in CFEM proteins is a key challenge for future research, as it will clarify their contributions to multifunctional roles.

The functional diversity of Type II GPI-anchored proteins further illustrates this evolutionary plasticity: cell wall organization in *S. cerevisiae* and *A. fumigatus*, heme acquisition in *C. albicans* and *B. bassiana*, and plant immunity modulation in phytopathogens. Despite these advances, the mechanisms underlying their functional diversification remain poorly understood, and future research must address it as well as to clarify their roles in wall integrity and host pathogenicity. Recent studies have increasingly centered on CFEM-containing effector proteins in fungi-plant interactions, where these proteins play roles in both the induction of cell death and the suppression of immune response (**Figures 5A, C**). Protein interaction studies, including CFEM11 from *F. graminearum* with ZmWAK17 and CfEC12 from *C. fructicola* with MdNIMIN2, provide initial insights into potential host targets (**Figure 5C**). However, significant gaps remain in our understanding of how these effectors are activated after secretion. It is still unclear whether CFEM effectors undergo post-translational modifications, proteolytic cleavage, or redox changes to become active in the apoplast or host cytoplasm. Additionally, despite evidence that CFEM proteins function in specialized infection structures (e.g., haustoria or appressorium), their non-canonical secretion mechanisms and vesicle-trafficking networks remain undefined.

Finally, Pth11-like proteins are the least-studied group of CFEM-containing proteins, despite their recognized importance in the formation of infection structures (**Figure 5D**). Current knowledge is largely limited to the genus *Magnaporthe*, which restricts the possibility of broader comparative analyses between genera or classes. Although these proteins are classified as G-protein coupled receptors, it is unknown whether Pth11-like proteins perceive the same stimuli as other putative fungal GPCRs. Clarifying this distinction will be crucial for understanding the functional specialization of CFEM-containing GPCRs. In conclusion, CFEM domain-containing proteins are important for fungal physiology and pathogenicity, serving as key links between host interaction and cellular maintenance. Future studies on their structural diversity and novel mechanisms will be crucial for developing targeted agricultural control strategies.

## Supplementary material

Supplementary Figure 1 Analysis of sequence similarity of CFEM-containing proteins (Frickey and Andrei, 2004). CLANS clustering of 10,000 sequences retrieved from Pfam. Sequences are represented by black dots. Lines connecting the dots correspond to BLASTP values better than 1xE-20.
